# Role of plant growth-promoting bacteria (PGPB) in enhancing phenolic compounds biosynthesis and its relevance to abiotic stress tolerance in plants: a review

**DOI:** 10.1007/s10482-025-02130-8

**Published:** 2025-07-24

**Authors:** Zuzanna Jakubowska, Marcin Gradowski, Jakub Dobrzyński

**Affiliations:** 1 Institute of Technology and Life Sciences-National Research Institute, Falenty, 3 Hrabska Avenue, 05-090 Raszyn, Poland; 2 Department of Biochemistry and Microbiology, Institute of Biology, Warsaw University of Life Sciences—SGGW, Warsaw, Poland

**Keywords:** Flavonoids, Abiotic stress tolerance, Sustainable agriculture

## Abstract

Biofortification of plants using Plant Growth-Promoting Bacteria (PGPB) represents a promising strategy in sustainable agriculture. This paper discusses the PGPB action in the context of their impact on phenolic compounds biosynthesis and the prospects for their application in agriculture. So far, no review article has summarized the significance of PGPB in increasing phenolic compounds in plants. PGPB, such as *Pseudomonas*, *Bacillus*, and *Azospirillum*, promote plant growth by producing phytohormones, enhancing nutrient availability, and stimulating the biosynthesis of secondary metabolites through the activation of Induced Systemic Resistance (ISR). The activation of ISR (Induced Systemic Resistance) by PGPB stimulates the phenylpropanoid pathway, which is the primary biosynthetic route for polyphenolic compounds, including phenolic acids and flavonoids, in plants. Studies indicate that PGPB may increase phenolic compounds content from 9% to over 200%, while simultaneously improving antioxidant activity. Through the secretion of phenolic compounds, PGPB also can mitigate abiotic stresses such as drought, salinity and heavy metal contamination. Among the phenolic compounds whose production in various plant parts can be stimulated by PGPB are flavonoids, such as quercetin, procyanidin B1, EGCG, and catechin, and phenolic acids, including caffeic acid, ferulic acid, and chlorogenic acid. Advancements in omics research will enable a more precise investigation of the impact of PGPB, including endophytic bacteria, on the biosynthetic pathways of phenolic compounds. In the future, this will translate into improved efficiency in stimulating the production of these compounds. Nevertheless, even now, the use of PGPB offers a sustainable alternative to genetic engineering, reducing reliance on chemical inputs in agriculture.

## Introduction

**Fig. 1:**
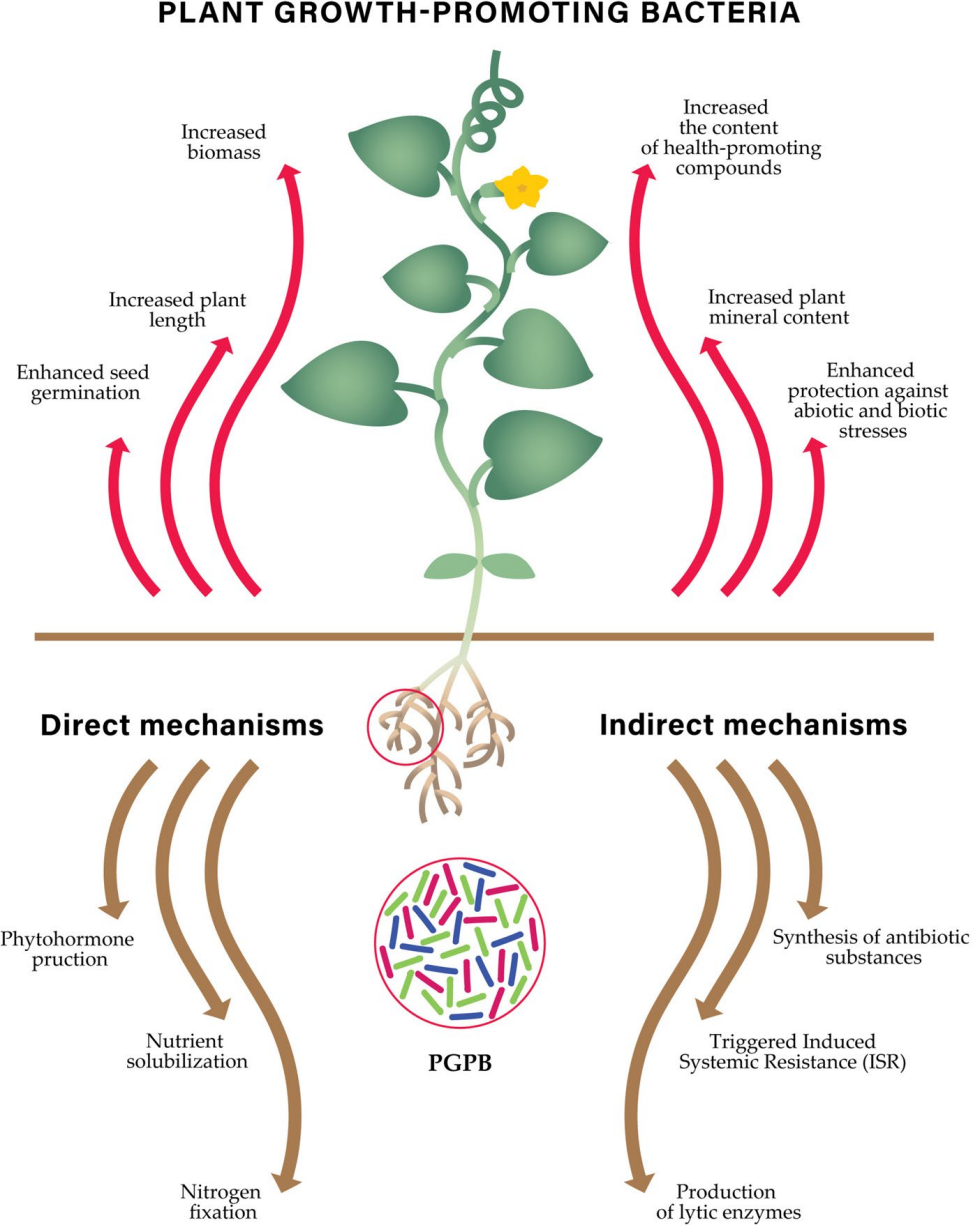
Plant Growth-Promoting Bacteria (PGPB) actions. The figure illustrates the effects and modes of action of the PGPB

Biofortification of plants, aimed at increasing the content of minerals, vitamins, and antioxidants, is gaining significant attention. Research on plant secondary metabolites, such as phenolic compounds, plays a key role in developing a new generation of crops (Gamelin et al. 2009). Nutritionally enhanced plants can be obtained through genetic manipulation or metabolism modification using elicitors. However, plant genetic engineering remains controversial among many consumers, and the introduction and cultivation of genetically modified plants are subject to strict legal regulations. Therefore, one of the most promising alternatives may be the application of Plant Growth-Promoting Bacteria (PGPB). PGPB are microorganisms naturally present in soil that positively influence plant growth and health (Scarano et al. 2020; Lü et al. 2024; Górska et al. 2024). Plant growth-promoting bacteria can stimulate plants both directly and indirectly [Figure 1]. Direct actions include the secretion of phytohormones (including auxins), nitrogen fixation (production of nitrogenase), and solubilization of phosphorus and potassium. Indirect actions are related to the mitigation of biotic and abiotic stresses, such as the stimulation of systemic resistance (ISR), production of fungistatic substances (lipopeptides, polyketides), and hydrolytic enzymes that break down the cell walls of pathogens and plant pests (Dobrzyński et al. 2025, 2024a, b, 2023; Dobrzyński and Naziębło 2024).Their use in agriculture aims to increase yields, reduce the reliance on chemical plant protection products, and support sustainable farming systems (Dobrzyński et al. 2025, 2023; Dobrzyński and Naziębło 2024). Importantly, some PGPB strains are capable of increasing the content of bioactive substances in plants including phenolic compounds. Phenolic compounds are among the most common and widely distributed bioactive compounds, characterized by a broad range of biological activities. They can be classified into two main groups: flavonoids (including subclasses such as flavones, flavanones, flavonols, flavanols, and isoflavones) and phenolic acids (divided into hydroxybenzoic and hydroxycinnamic acids) (Andrade et al. 2023). These secondary metabolites are essential for many plant functions and offer notable health benefits to humans (Abbas and Munawar 2017).Phenolic compounds perform a variety of crucial biological roles in plants. They act as antioxidants, neutralizing reactive oxygen species (ROS) generated during oxidative stress (Wollenweber and Dietz 1981; Belščak-Cvitanović, et al. 2018), and help alleviate abiotic and biotic stresses by regulating cellular signaling pathways and the expression of stress-defense-related genes (Belščak-Cvitanović, et al. 2018; Ouhaddou et al. 2023). Additionally, they serve as structural polymers in cell wall lignification and function as attractants—flavonoids and carotenoids influence the color of plant organs, aiding in pollinator attraction. Some phenolics, like flavonoids, act as UV filters, protecting tissues from radiation damage, while others, such as salicylic acid, play signaling roles in defense mechanisms. They also contribute to plant defense—tannins and phytoalexins inhibit pathogen and pest development (Belščak-Cvitanović, et al. 2018; Ouhaddou et al. 2023; Kumar et al. 2020).

This review will explore the interactions between bacteria and plants, with a particular focus on the role of PGPB in enhancing the content of phenolic compounds in plants. Special emphasis will be placed on their ability to stimulate the production of flavonoids and phenolic acids, as well as the mechanisms of their biosynthesis in response to PGPB. Additionally, gaps in the literature will be identified, and directions for future research will be proposed.

## Enhancement of phenolic compound production under normal conditions

### Mechanisms of activation of phenolic compound biosynthesis

PGPB modulate the content of phenolic compounds in plants through various mechanisms. Firstly, they may stimulate or inhibit the production of phytohormones, such as auxins or cytokinins, which influence plant metabolism, including phenol biosynthesis (Baxter et al. 2014; Sosnowski et al. 2023). Secondly, to a lesser extent, e.g. endophytic PGPB (including *Rhizobium laguerreae* HUTR05) may increase the content of phenolic compounds by secreting them into plant tissues (Baque et al. 2010). However, the primary mechanism by which PGPB affects phenolic compound biosynthesis is based on triggering Induced Systemic Resistance (ISR). Bacteria can induce ISR in plants by secreting various metabolites, including lipopeptides, siderophores, antibiotics, lipopolysaccharides or volatile organic compounds (VOCs) (Dobrzyński et al. 2023; Ayuso-Calles et al. 2020).

**Fig. 2:**
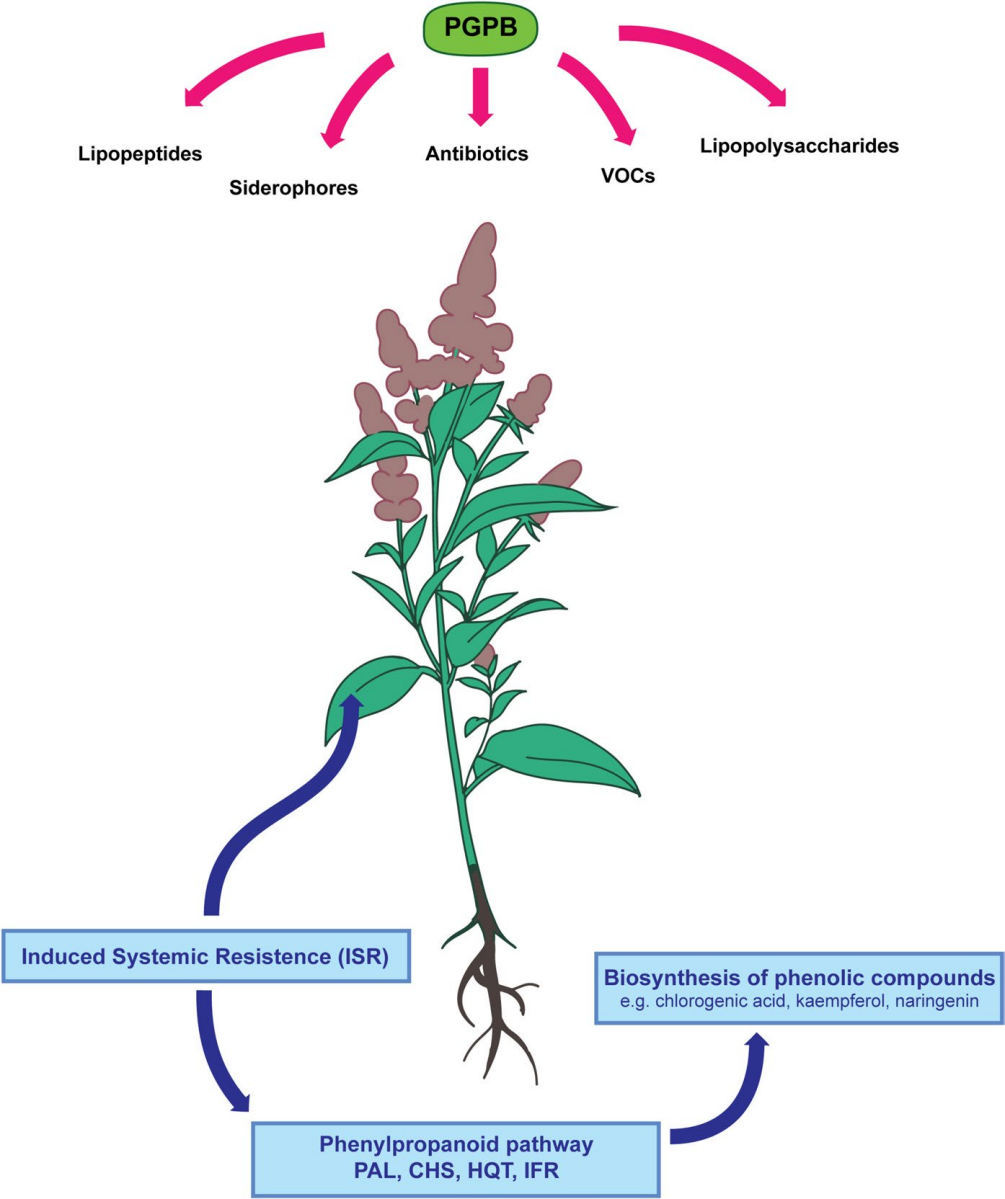
The mechanism of phenolic compounds biosynthesis in response to PGPB application

This process predominantly occurs through jasmonic acid (JA)-dependent signaling pathways, with minor contributions from salicylic acid (SA) and ethylene (ET) pathways (Meena et al. 2020; Bhattacharyya and Jha 2012; Asghari et al. 2020). JA is a well-established regulator of flavonoid biosynthesis, stimulating the expression of key genes in the phenylpropanoid pathway, such as genes encoding PAL (phenylalanine ammonia-lyase)—converts phenylalanine to cinnamic acid, which is precursor of various flavonoids (Kousar et al. 2020). Examples of genes encoding this enzyme whose expression is enhanced by PGPB are: *MtPAL2*, *MtPAL3* and *MtPAL4* (Khanna et al. 2019). PGPB can also increase the expression of *CHS* encoding chalcone synthase which catalyzes the initial step in flavonoid biosynthesis (forming chalcones) (Kisiel et al. 2024; El-Gendi et al. 2022).These compounds serve as precursors for flavonoids and other phenolic compounds. Additionally, plant growth-promoting bacteria can further enhance the expression of genes such as: i) *HQT*, which encodes hydroxycinnamoyl-CoA:quinate hydroxycinnamoyl transferase, an enzyme involved in the biosynthesis of chlorogenic acid (Kisiel et al. 2024; Zhao et al. 2022); ii) *IRF* gene encodes isoflavone reductase (IFR), an enzyme that specifically recognizes isoflavones and catalyzes a NADPH-dependent reduction to (3R)-isoflavanones (D’Orso et al. 2023; Wang et al. 2006). The mechanism of phenolic compounds biosynthesis in response to PGPB is illustrated in Figure 2.

Numerous studies have documented that plant growth-promoting bacteria can enhance the synthesis of various phenolic compounds in different plant tissues, such as: i) flavonoids: catechin, naringenin, naringin, myricetin, procyanidin B1, epigallocatechin gallate (EGCG), kaempferol, quercetin, K-3-O-rutinoside, Q-3-O-rutinoside, Q-3-O-glucoside, Q-3-O-glucuronide, kaempferol 3-O-glucuronide; ii) phenolic acids: gallic acid, protocatechuic acid, caffeic acid, ferulic acid, cinnamic acid, coumaric acid, chlorogenic acid, 4-hydroxy-2-methylacetophenone, 2,4-di-tert-butyl phenol; iii) other phenolic compounds: derivatives of caffeoyl acid, 5-O-caffeoylquinic acid, 4-methoxy-cinnamic acid hexoside, phloracetophenone (PHC) 4′-O-glucoside (Baque et al. 2010; Asghari et al. 2020; El-Gendi et al. 2022; Wiggins et al. 2022; Singh et al. 2003; Lavania et al. 2006; Bahadur et al. 2007; Jiménez-Gómez et al. 2021; Lobato-Ureche et al. 2023; Jagtap et al. 2023; Ferchichi et al. 2020)

### The genus *Pseudomonas*

The application of Plant Growth-Promoting Bacteria (PGPB), such as *Pseudomonas*, *Bacillus*, and *Azospirillum*, plays a significant role in enhancing the biosynthesis of phenolic compounds including flavonoids in plants. To date, several instances have been documented where inoculation with Pseudomonas bacteria altered phenolic compound profiles in plants. *Pseudomonas* sp. 42P4 was shown to enhance production of various phenolic compounds (mainly flavonoids) in pepper (Capsicum annuum L.) leaves, including increased levels of catechin, naringenin, myricetin, procyanidin B1, epigallocatechin-gallate, as well as cinnamic and ferulic acids (Lobato-Ureche et al. 2023). Furthermore, inoculation with a consortium of two strains—*P. fluorescens* Pf4 and *P. aeruginosa* Pag–resulted in elevated levels of phenolic acids like gallic, chlorogenic, and cinnamic acids in chickpea (*Cicer arietinum* L.) leaves. Singh et al. (Wiggins et al. 2022). In subsequent studies, this same consortium was also found to stimulate synthesis of gallic and ferulic acids in pea (*Pisum sativum* L.) leaves (Lavania et al. 2006). However, most studies report only the general impact on total phenolic compounds or total flavonoids content, without detailed identification of specific metabolites. For instance, Tariq et al. (Lobato-Ureche et al. 2021) evaluated the effects of *Pseudomonas putida* and *Pseudomonas stutzeri* strains on the growth and biochemical composition of garlic (*Allium sativum*). Their findings demonstrated that PGPB inoculation increased flavonoid content by 129% and phenolic compounds content by 263%. Additionally, seed germination rates, leaf and root growth, and both fresh and dry plant biomass showed notable improvement. Similar results were reported by Pellegrini et al. (Tariq et al. 2023), who utilized a bacterial consortium containing *Pseudomonas* spp. Their study observed a 25% increase in phenolic content and a 20% rise in antioxidant activity in onion (*Allium cepa L.*). Furthermore, inoculation enhanced plant growth, height, yield, and positively influenced the soil microbiological structure. In another study, Pérez-García et al. (Pellegrini et al. 2021) investigated the effects of *Pseudomonas paralactis* and *Bacillus cereus* consortia on phenolic compounds synthesis in cucumbers (*Cucumis sativus*). The results indicated a 9% increase in phenolic content and a 29% improvement in antioxidant activity, highlighting the beneficial effects of these bacterial consortia on the biochemical quality of plants. There are also studies in which the authors have obtained opposite relationships. Li et al. (Pérez-García et al. 2023) conducted an experiment on (marginal soils contaminated with heavy metals) and revealed that the *Pseudomonas* sp. TLC 6-6.5-4 caused the decrease in total phenolics, but significantly enhanced maize (Z*ea mays* L.) biomass, improved copper and phosphorus uptake efficiency, and mitigated oxidative stress.

### The genus *Bacillus*

Strains of the Bacillus genus may also play a significant role in enhancing plant quality and stimulating the biosynthesis of secondary metabolites, including inducing modifications in phenolic compound profiles. For instance, application of *Bacillus halotolerans* in coriander (*Coriandrum sativum L.*) cultivation led to a significant increase in the content of numerous phenolic compounds, including 5-O-caffeoylquinic acid, cinnamic acid, 4-methoxy-cinnamic acid hexoside, kaempferol-3-O-rutinoside, quercetin-3-O-rutinoside (rutin), quercetin-3-O-glucoside, and quercetin-3-O-glucuronide (Li et al. 2014). In similar studies, Jiménez-Gómez et al. (Bahadur et al. 2007) demonstrated that *Bacillus halotolerans* in a consortium with *Rhizobium laguerreae* significantly increased the content of protocatechuic acid, caffeic acid, and kaempferol 3-O-glucuronide in the leaves of endive (*Cichorium endivia* L.).

Furthermore, El-Gendi et al. (Kisiel et al. 2024) demonstrated that inoculation with *Bacillus subtilis* HA1-CF upregulated the expression of key genes involved in phenolic biosynthesis - *PAL*, *CHS*, and *HQT* - which consequently led to a 27% increase in total phenolic content and a 50% enhancement in flavonoid content in tomatoes. Similarly, Zhao et al. (El-Gendi et al. 2022) further highlighted the potential of the *Bacillus* sp. wp-6, which significantly stimulated the expression of the *CHS2* gene. This activity led to the production of PHC 4′-O-glucoside as a biosynthesis product. Additionally, *Bacillus cereus* induced the activity of enzymes such as PAL (phenylalanine ammonia-lyase) and POD (peroxidase), likely resulting in a 48% increase in phenolic compounds in the leaves of *Allium sativum* L. A similar rise in total phenolic content was observed with other bacterial strains, including *Bacillus subtilis* CBR05 [43-Chandra et al.] and *Bacillus safensis* FV46 (Chandrasekaran et al. 2019) in tomato plants (*Solanum lycopersicum* L.), as well as *Bacillus sphaericus* UPMB10 in banana plants (Costa-Santos et al. 2021). *Bacillus cereus* ATCC14579 in garlic leaves (*Allium sativum* L*.)* (Lobato-Ureche et al. 2021).

### The genus *Azospirillum* and other bacteria

Research on *Azospirillum* strains has also explored their influence on phenolic compounds, though fewer studies have been conducted compared to those involving *Pseudomonas* and *Bacillus* strains. Pellegrini et al. (Tariq et al. 2023) reported that a consortium containing *Azospirillum brasilense* increased phenolic compounds content in onions by 25%, enhanced antioxidant activity, and significantly boosted crop yield. Similarly, Consentino et al. (Maziah et al. 2010) evaluated the effects of *Azospirillum brasilense* DSM 1690 and DSM 2298 strains on phenolic compounds and flavonoids content in lettuce (*Lactuca sativa L.*). The highest levels of these compounds were observed in plants treated with moderate nitrogen doses (30–60 kg/ha) in combination with bacterial inoculation. However, excessive nitrogen application was found to inhibit bacterial activity.

In addition to the positive impact on the phenolic compound content in plants through inoculation with bacteria from the *Pseudomonas*, *Bacillus*, and *Azospirillum* genera, the influence on this group of compounds has also been documented when using bacteria from genera such as *Sinorhizobium*, *Herbaspirillum*, and *Pseudarthrobacter*. For example, the application of *Herbaspirillum seropedicae* in rice (*Oryza sativa* L.) cultivation resulted in the upregulated expression of isoflavone reductase in rice roots (catalyzes the biosynthesis of isoflavonoids) (Wang et al. 2006). Besides, Ham et al. (Consentino et al. 2022) investigated the effects of the *Pseudarthrobacter* sp*.* NIBRBAC000502770 on the growth and flavonoid content of yellow avens (*Geum aleppicum*).

Indeed, the literature includes several examples of bacterial consortia positively influencing phenolic compound content in various plant parts. Lobato-Ureche et al. (Ferchichi et al. 2020) studied a consortium consisting of *Cellulosimicrobium* sp. 60I1 and *Azospirillum brasilense* Az3, noting an increase in phenolic acids (including chlorogenic, caffeic, and gallic acids) and flavonoids (such as kaempferol-3-glucoside and quercetin) in pepper (*Capsicum annuum* L.) seeds. Moreover, a consortium comprising *Azospirillum brasilense*, *Gluconacetobacter diazotrophicus*, *Herbaspirillum seropedicae*, and *Burkholderia ambifaria* stimulated phenolic compound production (by 25%) in onion (*Allium cepa* L.) tubers (Tariq et al. 2023). Other consortia have also enhanced phenolic content in plants. For example, a consortium of *Bacillus cereus* (KBEndo4P6), *Acinetobacter radioresistens* (KBEndo3P1), *Pseudomonas paralactis* (KBEndo6P7), and *Sinorhizobium meliloti* (KBEkto9P6) increased phenolic content by 9% in cucumber (*Cucumis sativus* L.) shoots. Similarly, *Serratia nematodiphila* RGK and *Pseudomonas plecoglossicida* RGK boosted polyphenol and flavonoid levels across various plant species (Pellegrini et al. 2021).

The studies discussed above, along with additional research not detailed here, are summarized in Table 1.

**Table 1:** Comparison of different PGPB strains and their effects on plants

PGPB or Consortium	Plant	PGP traits	Results	References
*Pseudomonas putida* KX574857	*Allium sativum* L.	No data available	Increase in phenolic compounds content in 175% (tubers)	Lobato-Ureche et al. (2021)
*Pseudomonas stutzeri* Kx574858	*Allium sativum* L.	No data available	Increase in phenolic compounds content by 263% (leaves)	Lobato-Ureche et al. (2021)
*Pseudomonas paralactis* KBEndo6P7	*Cucumis sativus* L.	IAA production	Increase in phenolic compounds content by 5%.(shoots)	Pellegrini et al. (2021)
*Pseudomonas* sp. DSM 25356	*Lactuca sativa* L.	No data available	Increase in phenolic compounds content (leaves)	Maziah et al. (2010)
*Pseudomonas* sp. 42P4	*Capsicum annuum* L.	IAA and siderophores production, fix nitrogen and solubilize phosphate	Modified the phenolic compound profile, increasing catechin, naringin, naringenin, myricetin, procyanidin B1, epigallocatechin-gallate, cinnamic, and ferulic acids (leaves)	Jiménez-Gómez et al. (2021)
*Pseudomonas fluorescens* Pf4 *and P. aeruginosa* Pag	*Pisum sativum* L.	No data available	Induced accumulation of gallic and ferulic acids (leaves)	Lavania et al. (2006)
*Pseudomonas fluorescens* Pf4 *and P. aeruginosa* Pag	*Cicer arietinum* L.	Induced systemic resistance via SA-dependent pathway	Increase in contents of phenolic acids: gallic, chlorogenic, cinnamic (leaves); and increased the total phenolic compounds (leaves)	Wiggins et al. (2022)
*Pseudomonas fluorescens* and *Microccucuce yunnanensis*	*Cydonia oblonga* L.	Siderophores production	Increase in the phenolic compounds in the quince seedlings (roots)	Ham et al. (2022)
*Pseudomonas plecoglossicida* RGK and *Serratia nematodiphila* RGK	*Curcuma longa* L.	No data available	Increase in two phenolic compounds: 4-Hydroxy-2-methylacetophenone, 2,4-Di-tertbutyl phenol (rhizomes)	Lobato-Ureche et al. (2023)
*Pseudomonas brassicacearum* KK5*, P. corrugata* KK7*, and Paenibacillus borealis* KK4 and the symbiotic strain *Sinorhizobium meliloti* KK13	*Medicago truncatula* L.	No data available	Stimulating effect on changes in the expression of genes encoding PAL, in particular, MtPAL2, MtPAL3, and MtPAL4, on the activity of phenylalanine ammonia-lyase and on the total content of phenolic compounds (leaves)	Khanna et al. (2019)
*Pseudomonas fluorescens* WCS417r*, P. putida* SJ04*,* and *Bacillus subtilis* GB03	*Mentha piperita* L.	No data available	Increase in phenolic compounds content by 40–60% (leaves)	Rahimi et al. (2020)
*Bacillus halotolerans*	*Coriandrum sativum* L.	No data available	Enhance the contents of 5-O-caffeoylquinic acid, cinnamic acid, 4-methoxy-cinnamic acid hexoside, K-3-O rutinoside, Q-3-O-rutinoside, Q-3-O-glucoside and Q-3-O-glucuronide (leaves)	Li et al. (2014)
*Bacillus halotolerans* and *Rhizobium laguerreae*	*Cichorium endivia* L.	No data available	Increase in protocatechuic acid, caffeic acid and kaempferol 3-O-glucuronide contents (leaves)	Bahadur et al. (2007)
*Bacillus* sp. wp-6	*Tritucum aestivum* L.	IAA, siderophores production and solubilize phosphorus	Increase in biosynthesis of flavonoids including upregulation of chalcone synthase 2 and PHC 4′-O-glucoside (leaves)	El-Gendi et al. (2022)
*Bacillus subtilis* HA1-CF	*Solanum lycopersicum* L.	No data available	Induction of genes (PAL, CHS, HQT); increase in phenolic compounds by 27% and flavonoids by 50%. (leaves)	Kisiel et al. (2024)
*Bacillus subtilis* CBR05	*Solanum lycopersicum* L.	No data available	Increase in phenolic compounds content including flavonoids (fruits)	Jiménez-Gómez et al. (2020)
*Bacillus safensis* FV46	*Solanum lycopersicum* L.	IAA production	Increase in phenolic compounds content	Chandrasekaran et al. (2019)
*Bacillus sphaericus* UPMB10	*Musa* L.	No data available	Increase in phenolic compounds content (banana plantlets)	Costa-Santos et al. (2021)
*Paenibacillus glycaniliticus*	*Lupinus luteus* L.	No data available	not affect total phenolic content, but induce the accumulation of kaempferol (seeds)	Jagtap et al. (2023)
*Azospirillum brasilense*	*Oryza sativa* L.	No data available	isoflavone reductase was upregulated in expression in rice (roots)	Wang et al. (2006)
*Azospirillum brasilense* DSM 1690 and *Azospirillum brasilense* DSM 2298	*Lactuca sativa* L.	Nitrogen fixation	ncrease in phenolic compounds content (leaves)	Maziah et al. (2010)
*Herbaspirillum seropedicae*	*Oryza sativa* L.	No data available	Isoflavone reductase was upregulated in expression in rice (roots)	Wang et al. (2006)
*Pseudarthrobacter* sp. NIBRBAC000502770	*Geum aleppicum* L.	IAA production and activation of ISR	Increase in phenolic content by 3.1 times (shoots)	Ham et al. (2022)
*Cellulosimicrobium* 60I1,*Pseudomonas* 42P4	*Capsicum annum* L.	siderophores production and solubilize phosphorus	Increase in phenolic content (seeds)	Ferchichi et al. (2020)
Consortium: *Azospirillum brasilense, Gluconacetobacter diazotrophicus, Herbaspirillum seropedicae, Burkholderia ambifaria*	*Allium cepa* L.	No data available	Increase in phenolic compounds content (tubers) by 25%	Tariq et al. (2023)
Consortium: *Bacillus cereus* KBEndo4P6, *Acinetobacter radioresistens* KBEndo3P1, *Pseudomonas paralactis* KBEndo6P7, and *Sinorhizobium meliloti* KBEkto9P6	*Cucumis sativus* L.	IAA production	Increase in the total phenolic comounds by 9% (shoots)	Pellegrini et al. (2021)
*Bacillus subtilis, Bacillus megaterium*, and *Pseudomonas fluorescens*	*Lactuca sativa* L. var. crispa	No data available	Increase in total phenolic and flavonoid contents (leaves)	Rosario Cappellari et al. (2017)

## Role of PGPB in the biosynthesis of bioactive compounds and resistance to abiotic and biotic stresses

Abiotic stresses such as drought, salinity, and environmental pollution induce excessive production of reactive oxygen species (ROS) in plants, leading to oxidative stress. However, PGPB can mitigate these effects by stimulating the biosynthesis of phenolic compounds, which act as antioxidants to neutralize ROS. Among the strongest antioxidants whose production can be stimulated by PGPB are flavonoids such as quercetin, procyanidin B1, EGCG, and catechin, as well as phenolic acids, including caffeic acid, ferulic acid, and chlorogenic acid (Baque et al. 2010; Meena et al. 2020; Asghari et al. 2020; El-Gendi et al. 2022; Singh et al. 2003; Lavania et al. 2006; Bahadur et al. 2007; Jiménez-Gómez et al. 2021; Lobato-Ureche et al. 2023; Ferchichi et al. 2020).The following subsections discuss studies demonstrating the stimulation of phenolic compound biosynthesis under abiotic stress conditions such as drought, salinity, and heavy metal contamination.

### Drought stress

Drought stress is a critical environmental challenge that significantly impacts agriculture worldwide (Ikiz et al. 2024). Water scarcity, driven by climate change and natural weather fluctuations, degrades soil quality, and reduces biodiversity, ultimately destabilizing ecosystem balance (Yousaf et al. 2022; Takahashi et al. 2020; Rao et al. 2006; Salehi-Lisar and Bakhshayeshan-Agdam 2016). From an agricultural and horticultural perspective, one of the major problems associated with drought is the disruption of plant growth and development. A potential solution that may help mitigate the effects of drought in plants is the use of plant growth-promoting bacteria (Ahluwalia et al. 2021; Lucas et al. 2023). One of the key mechanisms for alleviating drought stress is the triggered production of secondary metabolites, such as flavonoids and polyphenols. Unfortunately, there are very few studies in the literature describing the positive effects of PGPB on the increase of phenolic compounds in plants under drought stress. For example, under drought conditions, the strain *Pseudomonas* sp. N 5.12 increased the total content of phenolic compounds by 21.5% in the leaves of tomato (*Solanum lycopersicum* L.). Moreover, a consortium composed of *Pseudomonas putida* (RA) and *Paenibacillus lentimorbus* CHM12 increased the phenolic compound content in maize (*Zea mays* L.). In another case, *Cellulomonas pakistanensis* (NCCP11) and *Sphingobacterium pakistanensis* (NCCP246) enhanced the same parameter in the roots of faba bean (*Vicia faba* L.). Interestingly, Tanveer et al. (Lucas et al. 2023) demonstrated that combining *Pseudomonas putida* with salicylic acid significantly increased flavonoid (39%) and phenolic compounds (40%) content in rapeseed plants. This increase was associated with improved drought tolerance and heightened activity of antioxidant enzymes, including superoxide dismutase (SOD), ascorbate peroxidase (APX), and catalase (CAT). These enzymes effectively mitigated oxidative stress, enhancing plant survival under challenging environmental conditions.

In summary, the use of PGPB, such as *Pseudomonas putida*, in combination with other biostimulants, presents an effective strategy for mitigating drought stress. These approaches enhance plant growth and resilience by increasing the accumulation of phenolic compounds, and other secondary metabolites while improving physiological parameters, such as photosynthesis and gas exchange. Consequently, plants exhibit greater adaptability to water scarcity and other environmental stresses.

### Salinity stress

Salinity stress poses a significant global challenge to agriculture, leading to soil degradation, reduced biodiversity, and substantial crop yield losses. Excessive salt accumulation in soil, primarily caused by improper irrigation and poor soil management, negatively impacts plant growth (Tanveer et al. 2023; Roy and Chowdhury 2021; Ondrasek et al. 2022). One of the key mechanisms for mitigating salinity stress is the enhanced production of secondary metabolites, such as flavonoids, polyphenols, and other bioactive compounds. Under saline conditions, plant growth-promoting bacteria (PGPB) can be used to increase the levels of phenolic compounds. For example, inoculation with *Rhizobium laguerreae* led to an increase in the content of compounds such as derivatives of caffeoyl acid and quercetin, which are antioxidants (Baque et al. 2010).

However, most studies in the literature only report the impact of PGPB on the overall quantity of phenolic compounds. Ali et al. (Parihar et al. 2015) demonstrated that inoculating maize with *Bacillus* sp*.* PM31 increased the production of phenolic compounds, thereby enhancing the plant's adaptability to saline conditions. The elevated biosynthesis of these metabolites improved antioxidant capacity, directly contributing to plant survival. Similar results were observed by Kang et al. (Ali et al. 2023), who studied the effects of *Acinetobacter pittii* YNA40, a strain capable of producing indole-3-acetic acid (IAA). In soybean, this strain significantly increased phenolic compounds and flavonoid levels.

Moreover, Redondo-Gómez et al. (Kang et al. 2023) reported that inoculation with PGPB from halophytes increased secondary metabolite content in Swiss chard leaves grown under 85 mmol L⁻^1^ NaCl salinity. Total phenolic compounds increased regardless of salinity levels, while inoculation restored flavonoid levels reduced by salinity. Anthocyanin content increased by 24% in salt-free plants after inoculation, though antioxidant capacity decreased under saline conditions regardless of treatment. These findings highlight the efficacy of PGPB in alleviating salinity stress. Similarly, Agha et al. (Redondo-Gómez et al. 2021) reported that bacterial inoculation significantly enhanced total phenolic compounds content in soybean (*Glycine max* L.) under both control and saline conditions. The combination of *Bradyrhizobium japonicum* and *Enterobacter* Delta PSK produced the highest phenolic compounds increase, indicating activation of plant defense mechanisms. Phenolic compounds levels in bacterially treated plants were significantly higher than in untreated controls, underscoring the role of PGPB in mitigating oxidative stress caused by salinity.

### Heavy metal stress

Heavy metal stress, induced by elements such as lead, cadmium, mercury, and arsenic, represents a significant global environmental concern. Heavy metals act as stressors for plants, disrupting physiological and biochemical processes (Agha et al. 2023; Noor et al. 2022; Pandey and Dubey 2019; Asati et al. 2016). As with other abiotic stresses, one of the key mechanisms by which plants mitigate the effects of heavy metal stress is the enhanced production of secondary metabolites, and PGPB can increase the production of, for example, phenolic compounds. Several studies in the literature confirm this. Khanna et al. (Kousar et al. 2020) reported that *Pseudomonas aeruginosa* and *Burkholderia gladioli* enhanced the expression of two key genes involved in phenolic compound biosynthesis, namely PAL and CHS, in cadmium (Cd)-stressed tomato (*Solanum lycopersicum* L.) seedlings. This upregulation resulted in increased production of phenolic compounds in the Cd-stressed seedlings. Furthermore, El-Ballat et al. (Patel, et al. 2021) demonstrated that inoculation with the *Azospirillum brasilense* EMCC1454 strain significantly increased the production of these compounds in chickpea plants exposed to chromium stress. Similarly, Sharma et al. (El-Ballat et al. 2023) reported that inoculating *Brassica juncea* plants with *Pseudomonas aeruginosa* and *Burkholderia gladioli* strains resulted in substantial increases in phenolic compounds and flavonoid contents. A 40% rise in phenolic compunds was observed in 40-day-old plants, and a 26.4% increase was recorded in 70-day-old plants. Additionally, higher levels of carbohydrates, trehalose, amino acids, and proline further mitigated chromium toxicity and promoted plant growth.

In conclusion, the application of PGPB, such as *Azospirillum brasilense*, *Pseudomonas aeruginosa*, *Burkholderia gladioli*, and *Bacillus* sp*.*, represents an effective strategy for mitigating the toxic effects of heavy metal stress. These bacteria enhance plant growth and health by increasing the accumulation of phenolic compounds, flavonoids, and other secondary metabolites, thereby improving plant resistance to adverse environmental conditions. Other studies on the involvement of PGPB in mitigating the effects of selected stresses by increasing the production of phenolic compounds are presented in Table 2.

**Table 2:** PGPB and their role in alleviating various abiotic stresses

PGPB	Plant	Stress	Results	References
*Bacillus* sp. Z2 and Z4 with arbuscular mycorrhizal fungi	*Chenopodium quinoa* L.	Drought	Increase in polyphenol and flavonoid contents (seeds)	Sharma et al. (2023)
*Bacillus sp., and Bacillus subtilis*	*Lactuca sativa* L.	Drought	Increase in flavonoid content and antioxidant activity (leaves)	Belščak-Cvitanović, et al. (2018)
*Pseudomonas* sp. N 5.12	*Solanum lycopersicum* L.	Drought	Increase in phenolic compounds by 21.5% (leaves)	Ahluwalia et al. (2021)
*Pseudomonas putida (RA) and Paenibacillus lentimorbus CHM12*	*Zea mays* L.	Drought	Increase in phenolic compounds (leaves)	Benaffari and Meddich 2023)
*Cellulomonas pakistanensis* (NCCP11) and *Sphingobacterium pakistanensis (*NCCP246)	*Vicia faba* L.	Drought	Increase in phenolic compounds content (roots)	Mishra et al. (2025)
*Azospirillum brasilense, Arthrobacter globiformis* *Burkholderia ambifaria, Herbaspirillum seropedicae, Pseudomonas* sp.	*Brassica napus* L.	Salinity	Increase in phenolic content (leaves)	Nafees et al. (2024)
*Brevibacterium casei* EB3 and *Pseudomonas oryzihabitans* RL18	*Salicornia europaea* L	Salinity	Increase in phenolic compounds content (aboveground biomass)	Rossi et al. (2021)
*Rhizobium laguerreae*	*Lactuca sativa* L.	Salinity	Increase in the content of certain phenolic acids and flavonoids, such as derivatives of caffeoyl acid and quercetin (leaves)	Baque et al. (2010)
*Providencia sp.* CR05 *Proteus mirabilis* TCR20	*Zea mays* L.	Heavy metal stress (chromium—Cr),combination of metallic (Cr) and drought stress	Increase in phenolic content (leaves)	Ferreira et al. (2024)
*Pseudomonas aeruginosa* and *Burkholderia gladioli*	*Solanum lycopersicum* L.	Heavy metal stress (Cd)	Increase in phenolic content and enhance gene expression of *CHS* and *PAL* (seedlings)	Kousar et al. (2020)

## Co-inoculation of plant growth-promoting microorganisms (PGPB) and arbuscular mycorrhizal fungi (AMF)

Interactions between PGPB and arbuscular mycorrhizal fungi (AMF) play a crucial role in stimulating the biosynthesis of secondary metabolites such as phenolic compounds, alkaloids, saponins, and tannins (Vishnupradeep et al. 2022). Studies by Benaffari et al. (Sharma et al. 2023) demonstrated that the application of AMF and PGPB, both individually and in combination (MR), significantly increased polyphenols and flavonoids content in quinoa seeds, enhancing their antioxidant properties. The combined treatment not only boosted dry seed weight by 25% but also reduced reactive oxygen species (H₂O₂) accumulation by 47%, due to increased antioxidant enzyme activity. These findings highlight the synergistic interactions between PGPB and AMF in enhancing plant tolerance to water stress.

Similar mechanisms were observed in a study by Ziane (Swamy et al. 2016), which revealed that AMF and PGPB consortia activated the phenylpropanoid pathway in date palms, increasing PAL activity and promoting the accumulation of polyphenols and lignin in roots. These changes reduced susceptibility to Fusarium wilt, suggesting that plant defense mechanisms can be effectively enhanced through microbial interactions. Furthermore, studies by Agnolucci et al. (Ziane et al. 2023) have shown that the diversity of AMF and PGPB influences the quantity and quality of bioactive compounds in plants, indicating the potential for manipulating these interactions to optimize their effects. Mixed inoculations of PGPB and AMF proved particularly effective, synergistically enhancing the uptake of minerals such as phosphorus, nitrogen, and potassium, while also boosting plant resistance through improved tolerance to biotic and abiotic stresses. Emmanuel and Babalola (Agnolucci et al. 2020) similarly demonstrated that co-application of AMF and PGPB under abiotic stress conditions, such as drought and salinity, stimulated phenolic compounds production in plant tissues. This increased secondary metabolite content resulted from the activation of biosynthetic pathways, significantly improving plant adaptability to environmental stresses.

Begum et al. (Emmanuel and Babalola 2020) further investigated the co-inoculation of arbuscular fungi (*Glomus versiforme*) and *Bacillus methylotrophicus* under drought stress in tobacco (*Nicotiana tabacum* L.). The results indicated that co-inoculation significantly increased flavonoid and phenolic compounds content in plants, with maximum increases of 71.74% and 57.85%, respectively, compared to control plants. These secondary metabolites were identified as key contributors to plant defense by mitigating oxidative damage and enhancing drought stress resistance.

These findings suggest that optimizing the co-inoculation strategies of PGPB and AMF in agriculture could involve selecting specific microbial strains that exhibit strong synergistic effects, tailoring inoculation protocols to different crop types and stress conditions, and fine-tuning application methods, such as seed coating, soil amendment, or foliar sprays. Such targeted approaches could maximize the benefits of these interactions, enhancing crop yield, stress tolerance, and the production of bioactive compounds, thereby promoting sustainable and resilient agricultural practices.

## Conclusions, challenges and risks

Phenolic compounds, including flavonoids, play a pivotal role in enhancing plant resistance to environmental stress. Acting as powerful antioxidants, they neutralize reactive oxygen species (ROS) and regulate signaling pathways as well as the expression of defense-related genes. The application of PGPB may significantly boost phenolic compounds production in plant tissues, improving their adaptability under unfavorable environmental conditions. The increased levels of these compounds not only enhance crop resilience but also improve crop quality and nutritional value, positioning PGPB as a promising tool in sustainable agriculture.

Despite these promising findings, relatively few studies have delved into the molecular mechanisms underlying the regulation of phenolic compounds and flavonoids biosynthesis. Further research is essential to elucidate how different PGPB strains influence the expression of genes involved in these processes, particularly those governing the production of phenolic acids and flavonoids in the various parts of plants. Advanced molecular biology tools could help elucidate the regulatory pathways involved in phenolic compound synthesis. For instance, the development of sequencing methods and bioinformatics tools in the context of metagenome-assembled genomes (MAGs) may aid in the discovery of endophytes or other bacteria involved in stimulating phenolic compound biosynthesis through induced systemic resistance (ISR) triggering. This knowledge would enable the development of innovative strategies for leveraging PGPB more effectively to improve crop quality, bolster resistance to environmental stress, and enhance the production of valuable metabolites such as phenolic acids and flavonoids.

Besides, among plant growth-promoting rhizobacteria, there may also be potential human and plant pathogens. To date, a number of bacterial species have been isolated that possess both desirable and undesirable traits including *Pseudomonas aeruginosa* (Begum et al. 2022), *Bacillus cereus* (Bakshi et al. 2023), *Pantoea agglomerans* (Kulkova et al. 2023), *Serratia marcescens* (Dutkiewicz et al. 2016), *Escherichia coli* (Kotoky et al. 2019). These bacteria could potentially be transmitted to food, posing a serious risk to human health. Therefore, researchers studying PGPB should consider including the detection of virulence genes in their isolates. Such an approach would allow for the assessment of risks associated with using these strains in agriculture, as well as preventing their uncontrolled spread in agricultural and food environments. Detection of these genes could also help identify strains that may pose a threat to food safety and public health.

Nevertheless, as previously noted, PGPB are a promising strategy for increasing the content of health-promoting compounds in plants. A holistic approach to research on them may lead to the development of effective and environmentally and human-safe biopreparations.

## Data Availability

No datasets were generated or analysed during the current study.
